# A Novel Compound C12 Inhibits Inflammatory Cytokine Production and Protects from Inflammatory Injury *In Vivo*

**DOI:** 10.1371/journal.pone.0024377

**Published:** 2011-09-08

**Authors:** Yi Wang, Congcong Yu, Yong Pan, Jianling Li, Yali Zhang, Faqing Ye, Shulin Yang, Hui Zhang, Xiaokun Li, Guang Liang

**Affiliations:** 1 Bioorganic and Medicinal Chemistry Research Center, School of Pharmaceutical Science, Wenzhou Medical College, Wenzhou, People's Republic of China; 2 Institute of Bioengineering, Nanjing University of Science and Technology, Nanjing, People's Republic of China; 3 Department of Pharmacy, The First Affiliated Hospital, Wenzhou Medical College, Wenzhou, People's Republic of China; 4 Norman Bethune College of Medical Science, Jilin University, Changchun, People's Republic of China; University of Hong Kong, China

## Abstract

Inflammation is a hallmark of many diseases. Although steroids and cyclooxygenase inhibitors are main anti-inflammatory therapeutical agents, they may cause serious side effects. Therefore, developing non-steroid anti-inflammatory agents is urgently needed. A novel hydrosoluble compound, C12 (2,6-bis(4-(3-(dimethylamino)-propoxy)benzylidene)cyclohexanone), has been designed and synthesized as an anti-inflammatory agent in our previous study. In the present study, we investigated whether C12 can affect inflammatory processes in vitro and in vivo. In mouse primary peritoneal macrophages, C12 potently inhibited the production of the proinflammatory gene expression including TNF-α, IL-1β, IL-6, iNOS, COX-2 and PGE synthase. The activity of C12 was partly dependent on inhibition of ERK/JNK (but p38) phosphorylation and NF-κB activation. In vivo, C12 suppressed proinflammatory cytokine production in plasma and liver, attenuated lung histopathology, and significantly reduced mortality in endotoxemic mice. In addition, the pre-treatment with C12 reduced the inflammatory pain in the acetic acid and formalin models and reduced the carrageenan-induced paw oedema and acetic acid-increased vascular permeability. Taken together, C12 has multiple anti-inflammatory effects. These findings, coupled with the low toxicity and hydrosolubility of C12, suggests that this agent may be useful in the treatment of inflammatory diseases.

## Introduction

Inflammation is a complex biological response. A number of reports have suggested that a chronic or acute inflammatory state is tightly associated with, and even constitutes a crucial part of the pathogenesis of a variety of diseases, including atherosclerosis [1], diabetic complications [2], obstructive pulmonary disease [3], asthma [4], arthritis [5], infectious diseases [6], and cancer [7]. In inflammatory diseases and ischemic processes, large amounts of cytokines are produced causing oedema, cellular metabolic stress, and finally, tissue necrosis [8]. Cytokines also induce vasodilatation and transient increase in capillary permeability producing extravasation of plasma proteins [9]. The proinflammatory cytokines tumor necrosis factor (TNF)-α, interleukin (IL)-1, and IL-6, and the proinflammatory enzymes cycloxygenase (COX)-2, prostaglandin E synthase (PGES) and inducible NO synthase (iNOS) which produce hormone-like mediators such as NO, are primarily involved in promoting inflammatory processes, and they also play an important role in those disorders [10], [11].

Steroids and cyclooxygenase inhibitors have long been used as the main therapeutic anti-inflammatory agents, but they are frequently associated with significant detrimental effects in patients [12], [13]. Thus, there is an urgent need for the development of unique anti-inflammatory agents. Non-steroid anti-inflammatory candidates targeting proinflammatory cytokines or inhibiting the overexpression of cytokines become a major focus of current drug development [14]. Our previous study has demonstrated that a novel compound, 2,6-bis(4-(3-(dimethylamino)-propoxy)benzylidene)cyclohexanone (C12), is able to dose-dependently inhibit LPS-induced TNF-α and IL-6 release in RAW264.7 macrophages [15]. In addition, C12 is a hydrosoluble compound when combined with HCl to form a quaternary ammonium salt. These *in vitro* advantages render C12 as a potential anti-inflammatory agent. The aim of the present work is to evaluate the anti-inflammatory effects of C12 and investigate its pharmacological mechanism in macrophages and mouse models. Herein, we showed that C12 inhibited ERK/JNK and NF-κB dependent inflammatory responses *in vitro*. The anti-inflammatory effects of C12 contribute to its increase of survival and multi-organic protection from endotoxin shock *in vivo*. These studies thus identify C12 as a unique anti-inflammatory agent that may be positioned as a therapeutic agent for the treatment of inflammatory diseases.

## Results

### C12 inhibited LPS-induced NO production in mouse peritoneal macrophages (MPMs)

**Figure 1:**
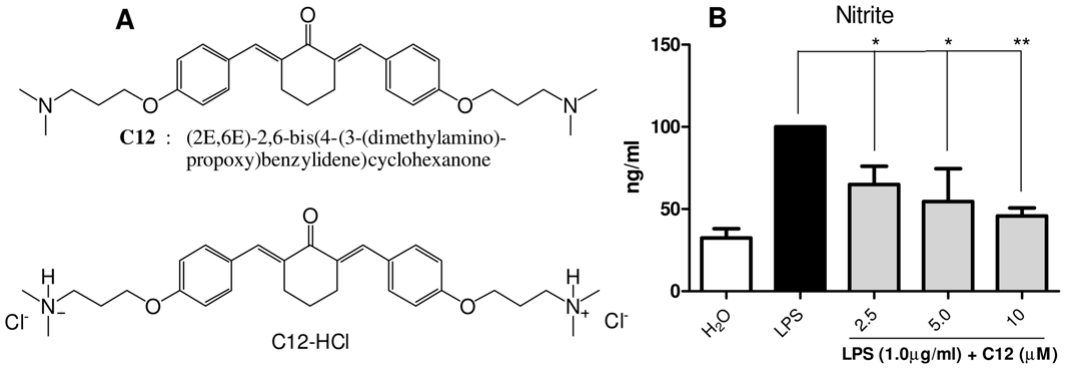
The structure and NO-inhibitory activity of C12. A. Chemical structures of C12 and its water-soluble form C12-HCl; B. C12 inhibitd nitrite production. MPMs were pretreated with vehicle (H_2_O) or C12 (2.5, 5, and 10 µM) for 2 h and then stimulated with 1 µg/mL LPS for 18 h. The nitrite levels in medium were detected as described in Materials and Methods. Bars represent the mean±SD of three independent experiments performed in duplicate, and asterisks indicate significant inhibition (* *p*<0.05, ** *p*<0.01).

Previously, we demonstrated that C12 was able to inhibit LPS-induced TNF-α and IL-6 release in a dose-dependent manner in RAW264.7 macrophages [15]. Due to the importance of NO as an inflammatory mediator on inflammation-related physiological and pathological processes [10], we tested the effect of C12 on the nitrite level, an index of NO production, in MPMs stimulated with LPS (1 µg/mL). As shown in Figure 1B, C12 dose-dependently repressed LPS-induced increase in nitrite after 18-hour incubation. C12 treatment at 10 µM reduced nitrite level by 45% compared to the LPS-challenged group (*p*<0.01).

### C12 suppressed LPS-induced proinflammatory gene expression in MPMs

**Figure 2:**
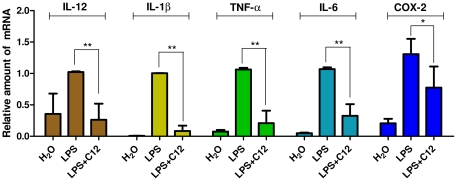
C12 inhibited LPS-induced inflammatory mRNA expression in MPMs. Cells were pretreated with compounds at 2.5 µM or vehicle (H_2_O) control for 2 h and treated with LPS (0.5 µg/mL) for 6 h. The mRNA levels of inflammatory cytokines IL-12, IL-1β, TNF-α, IL-6 and COX-2 were quantified by real-time quantitative PCR. The mRNA value was normalized to internal control β-actin mRNA and was expressed as a ratio to H_2_O. Bars represent the mean±SD of three independent experiments (* *p*<0.05, ** *p*<0.01).

MPMs were treated with LPS (0.5 µg/mL) for 6 h and examined for the expression of proinflammatory genes in the presence or absence of C12 by real-time quantitative PCR. In the dose-dependent study in our previous work [15], C12 at 2.5 uM exhibited an inhibition of 77.4% against LPS-induced IL-6 expression and an inhibition of 40.3% against TNF-α in macrophages. Therefore, we selected the concentration of 2.5 uM for further RT-qPCR study here. As shown in Figure 2, C12 at 2.5 µM potently inhibited LPS-induced up-regulation of IL-12 (74.3%, *p*<0.01), IL-1β (91.5%, *p*<0.01), TNF-α (80.2%, *p*<0.01), IL-6 (69.6%, *p*<0.01), and cycloxygenase-2 (COX-2, 40.7%, *p*<0.05) transcripts in MPMs.

### C12 inhibited LPS-induced NF-κB activation and ERK/JNK phosphorylation

**Figure 3:**
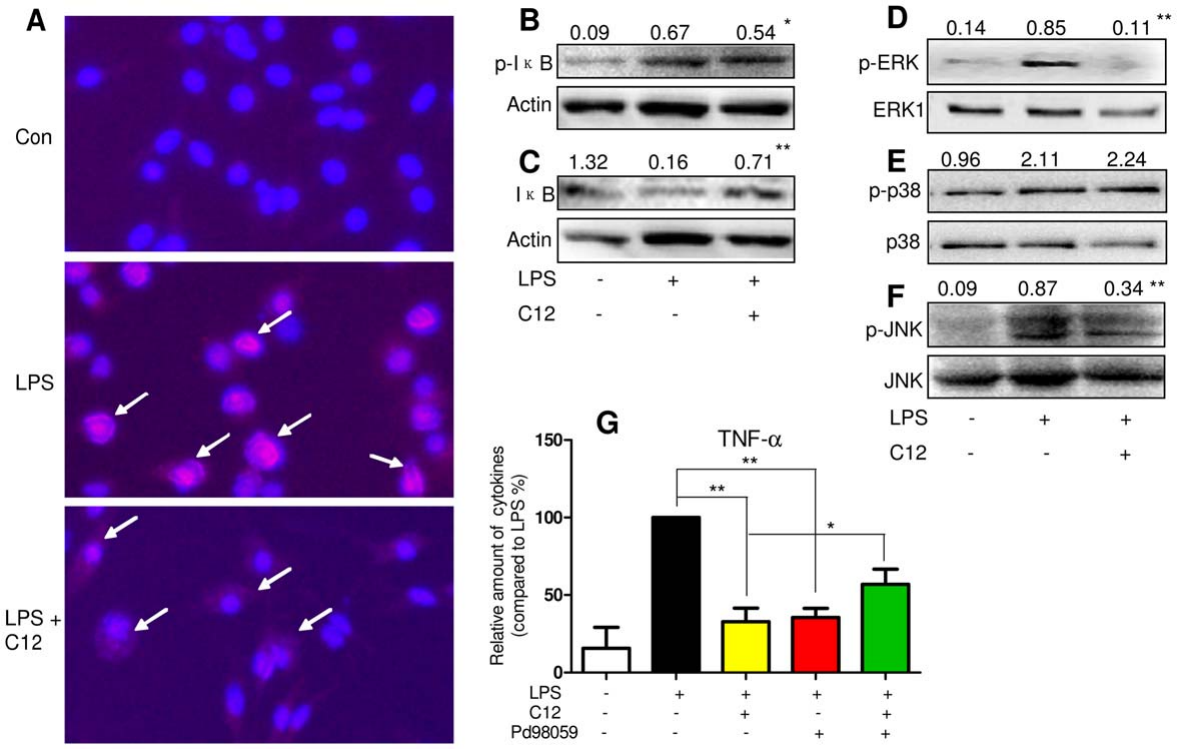
C12 inhibited LPS–induced NF-κB activation and MAPK phosphorylation. A. Cultured MPMs were pretreated with vehicle (H_2_O) or 10 µM C12 for 2 h and then stimulated with 0.5 µg/mL LPS. After 1 h of treatment, the cells were incubated with p65 antibody and Cy3 fluorescein-conjugated secondary antibody, and nuclei were stained with DAPI. The images (200×) were obtained by fluorescence microscope and overlay. Similar results were obtained with five independent experiments. B–F. MPMs were pretreated with vehicle (H_2_O) or C12 (10 µM) for 2 h followed by incubation with LPS (0.5 µg/mL) for 1 h. The protein levels of p-IκBα, IκBα, p-ERK, ERK, p-JNK, JNK, p-p38, and p38 were examined by western blot with actin as a loading control (values on the top of western blots represent the mean optical density ratio in three independent experiments, * *p*<0.05, ** *p*<0.01, *vs* LPS group). G. MPMs were pretreated with vehicle (H_2_O) or C12 (5 µM) for 2 h, and then stimulated with 0.5 µg/mL LPS for 12 h in the absence or presence of PD98059 (20 µM). The TNF-α level in medium were detected by ELISA as described in Materials and Methods. TNF-α expression is reported as the fold-expression relative to the LPS-treated group (n = 3, * *p*<0.05, ** *p*<0.01).

In the LPS-induced inflammatory stress, LPS-TLR4 signal transduction leads to MyD88-dependent phosphorylation of mitogen-activated protein kinases (MAPKs) and activation of nuclear factor (NF)-κB, which regulate the transcription of a variety of inflammatory factors and enzymes [16], [17]. We investigated whether C12 acts as an anti-inflammatory agent by inhibiting NF-κB and MAPKs. We first evaluated the effect of C12 on NF-κB p65 nuclear translocation. As shown in Figure 3A, LPS could strengthen NF-κB p65 nuclear translocation (red point in blue nucleus), while in C12-pretreated cells, LPS-induced nuclear p65 decreased, suggesting that C12, at a concentration of 10 µM, inhibited p65 translocation from cytoplasm to nuclei. IκBα phosphorylation and degradation frees NF-κB p65 from sequestration, allowing it to translocate to the nucleus, bind to target promoters, and turn-on transcription [12]. We next determined the total IκBα and phosphorylated IκBα in total cell protein extracts by Western blotting. LPS exposure for 1 h induced a 7.4-fold phosphorylation (Figure 3B) and an 87% degradation of IκBα (Figure 3C) in MPMs. Conversely, C12 at 10 µM inhibited LPS-induced IκBα phosphorylation (20% inhibition, Figure 3B) and degradation (4.43-fold increase, Figure 3C) in MPMs, respectively.

ERK, p38, and JNK constitute the MAPK family and are important intracellular components of the inflammatory responses to LPS. Especially, phosphorylated JNK and ERK could activate NF-κB signaling by IKKα/β-mediated IκBα phosphorylation and degradation [12], [16], [17]. Therefore, we tested whether the anti-inflammatory effect of C12 is due to its interference with these pathways. Figure 3D–3F shows that all of three MAPKs were activated by LPS stimulation. Among these kinases, the LPS-induced phosphorylation of ERK and JNK were significantly attenuated by pretreatment with C12 (10 µM). However, C12 did not decrease the amount of phosphorylated p38. Although C12 inhibits LPS-induced ERK phosphorylation in MPMs, it is unknown whether the ERK pathway is required for the inhibitory effects of C12 on proinflammatory responses. To this end, we utilized PD98059, a specific p-ERK inhibitor, to suppress the cellular ERK pathway activity. In the presence of PD98059, the inhibition of LPS-induced TNF-α release by C12 were significantly abrogated in MPMs (*p*<0.05, Figure 3G). These results imply that NF-κB activation and ERK phosphorylation might be an important step in mediating the anti-inflammatory effects of C12 in macrophages.

### C12 inhibited the increases of serum TNF-α, IL-6 and NO levels in LPS-challenged mice

**Figure 4:**
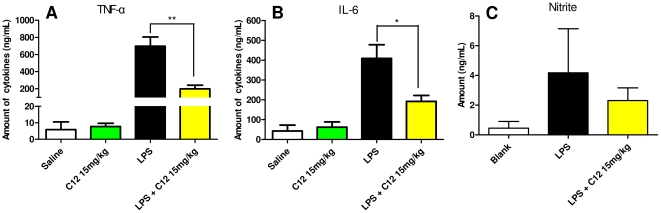
Inhibition of plasma TNF-α (A), IL-6 (B) and NO (C) by C12 in LPS-injected mice. C12 (15 mg/kg, i.v.) was given to B6 mice 15 min before LPS (10 mg/kg, i.v.) administration. Mice were killed and blood samples were collected 1 or 6 h after administration of LPS. Plasma TNF-α, IL-6 and NO concentrations were measured as described in Materials and Methods. Data point represent the mean±SD (n = 6). (* *p*<0.05, ** *p*<0.01, *vs* LPS group).

C12 was further evaluated its anti-inflammatory benefits in animals. Given the *in vitro* results and *in vivo* toxicity test in (Figure S1), mice were administered with C12 at 15 mg/kg (i.p.) or 10 and 30 mg/kg (i.v.) in the following *in vivo* experiments (Text S1). The serum levels of TNF-α, IL-6 and NO can reflect the degree of inflammatory response and injury *in vivo*. To evaluate the *in vivo* anti-inflammatory effect of C12, C57B/L6 (B6) mice administered with LPS (10 mg/kg, i.v.) and C12 (15 mg/kg, i.v.) were sacrificed and the plasma levels of inflammatory mediators were determined by ELISA. The plasma levels of TNF-α, IL-6 and NO increased over the 1–12 h period after LPS injection, and reached peak at 1 h (TNF-α), 6 h (IL-6) and 6 h (NO), respectively (data not shown). Pretreatment with C12 showed a 71.5% (n = 6, *p*<0.01) inhibition on TNF-α levels and a 53.0% (n = 6, *p*<0.05) inhibition on IL-6 levels compared to the LPS group (Figure 4A and 4B). Plasma nitrite level also increased at 6 h after LPS administration and pretreatment with C12-inhibited LPS-mediated NO production by 44.8% (Figure 4C, statistically non-significant between C12+LPS group *vs.* LPS group).

### C12 pretreatment reduced inflammatory gene expression in mouse liver after LPS challenge

**Figure 5:**
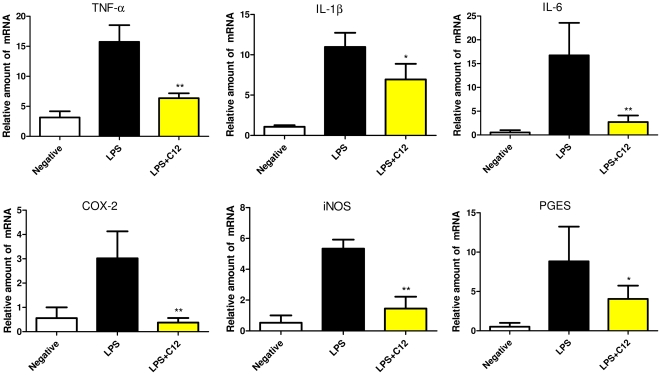
Inhibitory effects of C12 pretreatment (15 mg/kg, i.v., 15 min before LPS injection) on the induction of inflammatory gene mRNAs in B6 mouse livers 1 or 6 h after LPS administration (10 mg/kg, i.v.). Liver mRNA from vehicle control, LPS, and LPS+C12-treated mice was reverse transcribed for quantitative real-time PCR analysis of the expression of TNF-α, IL-6, IL-1β, COX-2, iNOS, and PGES. Expression is normalized to actin and reported as the fold-expression relative to one of samples in the vehicle control group. Values shown are the mean ± SD of 3 mice from the saline control group (white), and 4–6 mice each from the LPS (black)- and LPS+C12 (yellow)- treated groups. (* *p*<0.1, ** *p*<0.05, compared to LPS group).

Real-time qPCR was used to verify the profiles of selected genes TNF-α, IL-1β, IL-6, COX-2, iNOS and mPGES that were involved in inflammatory response in mouse liver after LPS challenge. A dose of LPS (10 mg/kg, i.v.) and C12 (15 mg/kg, i.v.) was chosen for these studies. The mRNA levels of these six genes in the liver were significantly induced by LPS administration, while they peaked at different time points (1 to 6 h), respectively (data not shown). TNF-α, IL-1β, and IL-6 were determined at 1 h, 3 h and 6 h after LPS injection, respectively. As illustrated in Figure 5, the hepatic mRNA expression of TNF-α, IL-1β, IL-6, COX-2, iNOS and mPGES was suppressed (59.6%, 36.6%, 83.8%, 87.5%, 72.8% and 54.1% inhibition, *p*<0.05 or *p*<0.01, respectively) in C12-pretreated mice compared with LPS-treated group. These results suggested that C12 significantly inhibits the LPS-induced increase of hepatic inflammatory gene transcription.

### Effects of C12 pretreatment on lung histology in mice after LPS administration

**Figure 6:**
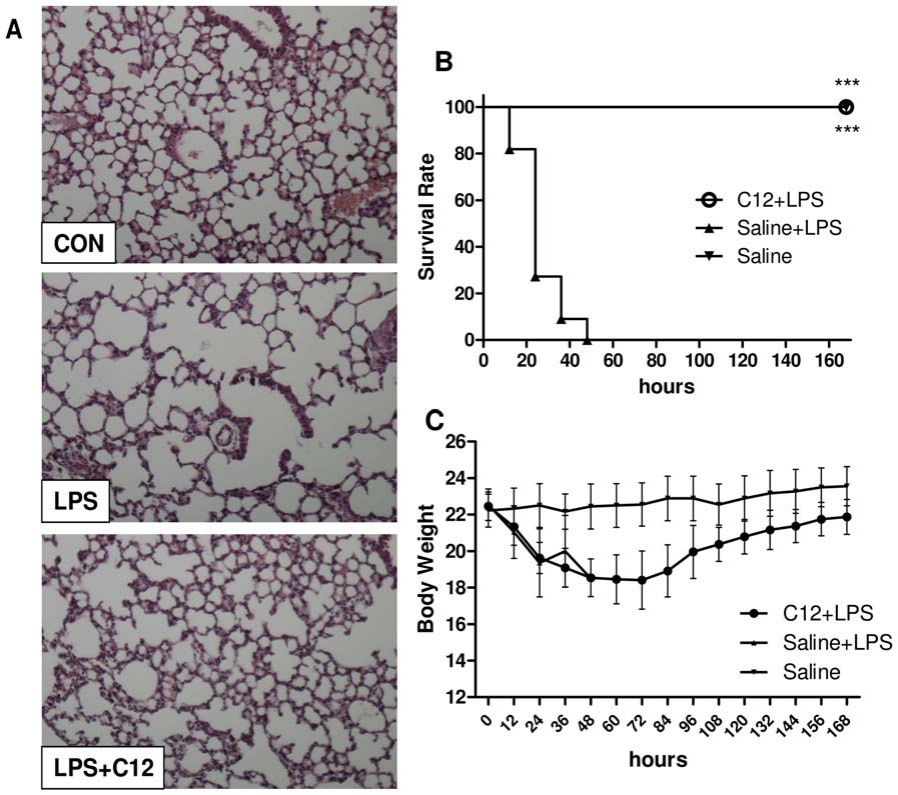
Beneficial Effects of C12 against acute inflammatory injury and shock in B6 mice. A. Mice were treated with C12 (15 mg/kg, i.v.) 15 min before LPS (10 mg/kg, i.v.) administration. After 48 h, lung histopathological analysis was performed using H&E staining as described in Materials and Methods (magnification ×200; n = 3; Con, control). B and C. C12 improves survival of mice subjected to a lethal dose of LPS. Mice were pretreated with vehicle (saline) or 15 mg/kg C12 (i.v.) 15 min before the injection of 20 mg/kg of LPS (i.v.). Survival (B) and body weight (C) were recorded for 7 days after the LPS injection at the interval of 12 h. Results from the summary of two different experiments are shown. n = 12 animals in each group. C12 improved survival rate at 12–48 h (*** p<0.0001).

Acute lung injury (ALI) is one of the most sensitive responses to LPS injection and ultimately leads to alveolar flooding with serum proteins and oedema fluid [18]. After mice were administrated LPS (10 mg/kg, i.v.), histological changes in the B6 mouse lung such as inflammatory cell infiltration, peri-vascular oedema, and marked alveolar hemorrhage, were observed by H&E staining. C12 pretreatment at 15 mg/kg improved such pulmonary damage remarkably (Figure 6A). C12 also amended the LPS-injured tissue structure of pulmonary lobules and LPS-induced expansion of lung alveolus.

### C12 treatment prolonged survival in an acute inflammatory model

To investigate the beneficial effects of C12 on acute inflammatory shock, we infected B6 mice with LPS (20 mg/kg, i.v.) and compared survival rates between C12-treated and placebo control mice. All C12-untreated control mice had died at 48 h after LPS injection, while all of C12-treated animals survived the acute phase of inflammation and displayed 100% survival rates until the end of the experiment (n = 12, *p*<0.0001, Figure 6B). Meanwhile, the body weight of C12-treated mice decreased during 0–48 h but regained slowly since by 60 h after LPS treatment (Figure 6C). Thus, C12 treatment prolongs survival in the acute inflammatory shock model.

### Treatment with C12 reduced carrageenan-induced paw oedema and inhibited inflammation-related reactions in chemically-induced inflammatory models

**Figure 7:**
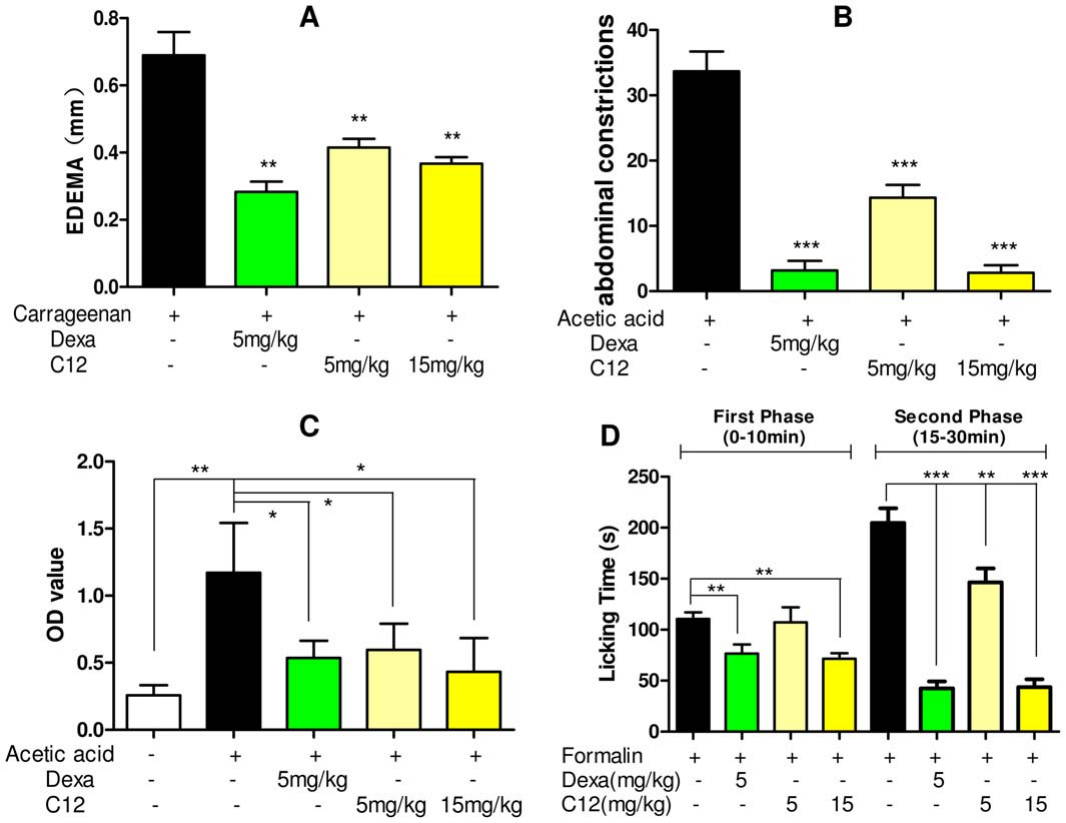
Effects of C12 (5 mg/kg or 15 mg/kg, i.p.) on the chemical-induced inflammatory response. A. Paw oedema models induced by carageenan; B. acetic acid-induced pain model; C. acetic acid-vascular permeability models; D. formalin-induced nociception mice (first-inflammatory phase, left; second-inflammatory phase, right). Control animals received saline (0.9% NaCl). Dexamethasone (5 mg/kg) was used as a positive control. Each value represents the mean±SD of 6 animals, and asterisks indicate significant difference of the paw thickness in relation to the corresponding saline group (* *p*<0.05, ** *p*<0.01, *** *p*<0.001).

We further tested the beneficial effects of C12 on inflammation-related models challenged by another stimulus with dexamethasone as a positive control. Paw oedema induced by carrageenan has been widely used as an inflammatory model *in vivo* [19], [20]. ICR mice were initially treated with C12 (5 and 15 mg/kg) by i.p. injection followed by the paw oedema induced by carrageenan (300 µg/paw) for 4 h. As shown in Figure 7A, compared to carrageenan group, pretreatment with C12 reduced the oedema in size in a dose-dependent way, with the inhibitory rate in oedema size of 39.9% (5 mg/kg, *p*<0.01, *v.s.* control) and 46.9% (15 mg/kg, *p*<0.01, *v.s.* control), respectively (Figure 7A). We also pretreated ICR mice with C12 (5 and 15 mg/kg, i.p.) 15 min before chemically-induced inflammatory constrictions with acetic acid (0.6%, 200 µL, i.p.). The inflammatory pain and mice writhing response induced by acetic acid were significantly inhibited by C12 pretreatment in a dose-dependent manner with inhibitory rate of 57.4% (*p*<0.001), and 91.6% (*p*<0.001), respectively (Figure 7B). Besides inflammatory gene expression, acetic acid-caused pain and carrageenan-caused paw oedema are usually related to the vascular permeability increasing the release of cytokines from blood vessels to the injured tissues [21]. To demonstrate if C12 can reduce permeability changes during inflammation, we performed a vascular permeability assay in ICR mice using azovan blue and acetic acid. As shown in Figure 7C, acetic acid increased the permeation of azovan blue from the vascellum to abdominal cavity (4.5-fold, *p*<0.01), while pre-treated with C12 can significantly reduce the OD value compared to the acetic acid-treated group (5 mg/kg, 49%, *p*<0.05; 15 mg/kg, 63%, *p*<0.05), suggesting that C12 is able to inhibit vascular permeability and reduce inflammatory effusion.

Moreover, the inhibitory effects of C12 on the formalin-induced inflammatory nociception (both first and second phase) in ICR mice were evaluated. As shown in Figure 7D, the results revealed that i.p. pretreatment of C12 could dose-dependently reduce the licking times of the mice stimulated by formalin, with an inhibition of 35.3% (15 mg/kg, *p*<0.01) in first phase (0–10 min after formalin injection) and an inhibition of 78.7% (15 mg/kg, *p *0.001) in second phase (15–30 min after formalin injection), respectively. These results potently suggested that C12 is able to effectively inhibit the *in vivo* inflammatory response induced by chamical stimulus such as carrageenan, acetic acid and formalin.

## Discussion

Our previous study has found that a novel compound C12 possessed inhibitory effects on LPS-induced TNF-α and IL-6 release in RAW264.7 macrophages [15]. With another advantage of water-solubility when combined with HCl, C12 has been considered a promising anti-inflammatory candidate. In the present study, we demonstrated anti-inflammatory activities of this compound both *in vitro* and *in vivo*, using LPS-stimulated MPMs and several mouse models of topical inflammation, respectively.

Macrophages play important roles in a host's immune defense system during infection as well as in the processes of disease development [22]. Activation of macrophages by stimuli, such as LPS, increases the production of numerous inflammatory mediators, including various cytokines and nitric oxide (NO) [10]. Excessive production of NO has been widely reported to be associated with inflammatory response [23], [24]. First, we showed that C12 has a dose-dependently inhibitory activity upon NO synthesis in MPMs (Figure 1B). Strong inhibition of inflammatory cytokine release has been ascribed to down-regulation of NF-κB-dependent gene transcription [25], [26]. Our next experiments provided the evidence that C12 significantly inhibits IL-12, IL-1β, TNF-α, IL-6 and COX-2 mRNA transcription in MPMs (Figure 2). In addition, our results shown in Figure 4 demonstrated that C12 significantly inhibits the plasma NO level and serum levels of TNF-α and IL-6 in LPS-stimulated mice. In response to LPS, plasma TNF-α increased at a relatively early stage (1 h after challenge) as a proinflammatory factor and then induced downstream inflammatory reactions, including IL-6 and iNOS expression (6 h after challenge) in a NF-κB-dependent manner.

Endotoxin, or more specifically LPS, is an inflammatory stimulus that can exert effects on major organs, such as the liver. Several groups have found that LPS could increase inflammatory gene expression in liver of mouse models [27], [28]). We further surmised that C12 would decrease LPS-induced inflammatory gene expression in liver. Figure 5 presented the evidence that i.v. injection of C12 significantly reduced the mRNA levels of a host of proinflammatory mediators (TNF-α, IL-1β, IL-6, COX-2, iNOS, and PGES) in the LPS-challenged mouse livers. Thus, C12 is able to decrease the production of proinflammatory mediators both *in vitro* and *in vivo*. LPS injection further causes a whole-body inflammatory response such as multiple organ failure (MOF), including lung injury. The pulmonary injury is considered to be a direct cause of death by LPS injection, and often occurred in the context of MOF [29]. The lung histological examinations in Figure 6A demonstrated that C12 suppressed LPS-caused severe lung damage remarkably. LPS-induced acute multi-organ failure ultimately causes animal death. We further confirmed that C12 would significantly decrease LPS-induced lethality. Thus, the shift toward an anti-inflammatory cytokine profile by C12 is in agreement with its beneficial effect in the endotoxemic shock model.

Of particular interest in this study is the identification of molecular mechanism by which C12 inhibits inflammatory gene expression. LPS binds to TLR4-CD14-MD88 complex on the cell surface to trigger MAP kinase phosphorylation and then activation of NF-κB, a transcription factor normally sequestered in the cytoplasm that upon stimulation translocates to the nucleus to drive transcription of target genes, including cytokines, chemokines and cellular adhesion molecules [12], [16], [17]). The transcription factor NF-κB is retained in the cytoplasm in an inactive form by the inhibitory protein IκB. Activation of NF-κB requires that IκB be phosphorylated on serines 32 and 36, which results in targeted degradation of IκB. The dissociation of IκB causes the translocation of NF-κB p65 sub-unit from the cytoplasm to nucleus. The latter binds to the DNA site and triggers the inflammatory gene expression. Herein, we showed that C12 inhibited LPS-induced ERK/JNK phosphorylation and NF-κB activation and the subsequent induction of proinflammatory mediators in MPMs (Figure 3). JNK has been demonstrated as an upstream regulator of NF-κB signal [12], [16], [17]. Although MAPK pathways including ERK, p38, and JNK are all involved in LPS-stimulated inflammation, we did not find that C12 affects p38 activation. Furthermore, the anti-inflammatory effect of C12 was attenuated by ERK-specific inhibitor, and PD98059 also significantly inhibited the LPS-induced TNF-α release (Figure 3G). These results indicate that ERK critically plays a key role to turn on proinflammatory gene expression in LPS-stimulated macrophages, and at least partly, C12 exerts an anti-inflammatory effect via inhibition of ERK pathway. The partial reversion of p-ERK inhibitor suggests that there exist ERK-independent mechanisms in the anti-inflammatory action of C12. In the other hand, the leading curcumin has been reported to exert anti-inflammatory effects by multi-targeting mechanisms and the direct binding targets of curcumin are still unknown. Therefore, although this work only focuses on the MAPK/NF-κB-mediated inflammation, further studies are necessary to establish such notion as examination of the underlying molecular mechanisms and direct molecular targets of C12.

Besides the endotoxin and etiologic agents, some chemicals also induce the production of inflammation. During tissue inflammation, there is normally vasodilatation and transient increases in capillary permeability producing extravasation of plasma proteins and tissue oedema. Generally, these reactions are linked to painful perception or hyperalgesic sensitization [19], [30], [31]. Our results shown in Figure 7 indicate that pre-treatment with C12 reduced the inflammatory pain in the acetic acid and formalin models, and also reduced the paw oedema induced by carrageenan, and C12 can inhibit plasma substance extravasation from the blood vessel. These inflammatory models are mainly related to local production of bradykinin and prostaglandins, such as PGE2 and leukotrienes, both derivatives of arachidonic acid. These prostanoids bind the prostaglandin sub-type receptors, triggering the inflammatory and hyperalgesic pathway in tissues. Bradykinins and related kinins represent a group of potent inflammatory mediator peptides involved in pain and inflammation. Bradykinin receptor B1 is associated with a metabotropic signaling pathway producing vasodilatation, an increase of vascular permeability, and an increase of eicosanoids production, such as PGE2 and NO produced by PGES, COX-2 and iNOS. On the other hand, B1 receptors promote pain stimuli and inflammation by the NF-κB pathway [32], [33]. Thus, the inhibitions of inflammatory enzyme expression (Figure 5) and NF-κB activation (Figure 3) may mainly contribute to these beneficial effects of C12. Of course, the results are also partly related with the effects of C12 on inflammatory cellular activation during the recruitment of leukocytes to an injured target. In another inflammatory model of formalin-caused pain, the pain in the first phase (0–10 min after formalin i.p. injection) is induced through formalin directly stimulating C-fibers, whereas in the second phase (15–30 min), the pain is generated by the release of several inflammatory mediators [30], [34]. Thus, the licking times of the mice in different intervals reflect the degree of the pain. The higher inhibitory rate of C12 in phase II than phase I also partly indicated that the main mechanism of C12 activity is to inhibit the production of inflammatory mediators.

Steroids and cyclooxygenase inhibitors have long been used as major therapeutical anti-inflammatory agents. However, they often cause serious side effects in patients [12], [13], [14]. Thus, developing unique anti-inflammatory agents is currently in high demand. C12 is a new anti-inflammatory compound designed and synthesized in our lab. In conclusion, C12 effectively suppressed the production of various inflammatory mediators at the levels of both protein and mRNA by suppressing the activation of ERK/JNK//NF-κB signaling. The evidence from *in vivo* studies also supports that, with the advantages of water-solubility and low toxicity (Figure S1 and Text S1), C12, as a non-steroidal anti-inflammatory candidate, could provide a therapeutic option in the treatment of topical inflammatory diseases.

## Materials and Methods

### Animals

Male Institute of Cancer Research (ICR) mice and C57BL/6 mice weighing 18–22 g were obtained from the Animal Center of Wenzhou Medical College (Wenzhou, China). Animals were housed at a constant room temperature with a 12∶12 hour light-dark cycle, and fed with a standard rodent diet and water. The animals were acclimatized to the laboratory for at least 3 days before use in experiments. Protocols involving the use of animals were approved by the Wenzhou Medical College Animal Policy and Welfare Committee (Approval documents: 2009/APWC/0031).

### Reagents

Lipopolysaccharide (LPS), carrageenan, acetic acid and formalin solution were purchased from Sigma (Louis, MO). Dexamethasone was purchased from Xianju Pharma (Zhejiang, China). Saline was prepared as 0.9% NaCl solution. C12 was synthesized and characterized as described in our previous publication [15], [35]. C12 was used in the form of C12-HCl in all of the following experiments. Briefly, C12-HCl (C12) was dissolved in H_2_O and the solution was filtered through 0.22 µm microporus filters (Carrigtwohill, Co.Cork, Ireland) before use. The structures of C12 and its chloride are shown in Figure 1.

### Primary cell preparation and culture

For peritoneal macrophage preparation, ICR mice were stimulated by intraperitoneal (i.p.) injection of 3 ml thioglycollate solution (Beef extract (0.3 g), tryptone (1 g), sodium chloride (0.5 g), and soluble starch (6 g) were dissolved and boiled in 100 ml water. Before used, the solution was filtrated with 0.22 µm filter.) per mouse and kept in pathogen-free conditions for 3 days before peritoneal macrophage isolation. Total peritoneal macrophages were harvested by washing the peritoneal cavity with PBS containing 30 mM of EDTA (8 mL per mouse), centrifuged, then the pellet was resuspended in RPMI-1640 medium (Gibco/BRL life Technologies, Eggenstein, Germany) with 10% FBS (Hyclone, Logan, UT), 100 U/mL penicillin, and 100 mg/mL streptomycin. Peritoneal macrophages were cultured on 60 mm plates (1.2×10^6^ cells in 3 ml media per plate) and maintained at 37°C in a 5% CO_2_-humidified air. Nonadherent cells were removed by washing with medium at 3 h after seeding. Experiments were undertaken after the cells adhered firmly to the culture plates.

### Nitrite assay

A quantity of 100 µL of cell culture supernatant (or plasma in animal experiments) was collected and combined with 50 µL 1% sulfanilamide in 5% H_3_PO_4_ and 50 µL 0.1% N-(1-naphthyl)ethylenediamine dihydrochloride (Sigma, Louis, MO) in water, in a 96-well plate, followed by spectrophotometric measurement at 550 nm, using a microplate reader. Nitrite concentration in the supernatant was determined by comparison with a sodium nitrite standard curve. Experiments were performed at least three times and in duplicate.

### Western Immunoblot analysis

The treated cells were collected and lysated, then 30 µg of the whole cell lysates were separated by 10% sodium dodecyl sulfate-polyacrylamide gel electrophoresis and electrotransferred to a nitrocellulose membrane. Each membrane was preincubated for 1 h at room temperature in Tris-buffered saline, pH 7.6, containing 0.05% tween 20 and 5% non-fat milk. Each nitrocellulose membrane was incubated with specific antibodies against p-p38, p38, p-ERK, ERK, p-JNK, JNK (Santa Cruz, CA), and p-IκBα, IκBα, Actin (Cell Signaling Technology, Danvers, MA). Immunoreactive bands were then detected by incubating with secondary antibody conjugated with horseradish peroxidase and visualized using enhanced chemiluminescence reagents (Bio-Rad, Hercules, CA).

### Assay of cellular NF-κB p-65 translocation

The cells were immunofluorescence-labeled according to the manufacturer's instructions using a Cellular NF-κB p-65 Translocation Kit (Beyotime Biotech, Nantong, China). Briefly, after washing and fixing, cells were incubated with a blocking buffer for 1 h to suppress non-specific binding. Next, cells were incubated with the primary NF-κB p65 antibody for 1 h, followed by incubation with a Cy3-conjugated secondary antibody for 1 h, then with DAPI for 5 min before observation. P65 protein and nuclei fluoresce red and blue, respectively, and can be simultaneously viewed by fluorescence microscope (Nikon, Tokyo, Japan) at an excitation wavelength of 350 nm for DAPI, and 540 nm for Cy3. To create a two-color image, the red and blue images were overlaid, producing purple fluorescence in areas of co-localization [36].

### Treatment of mice with C12 and LPS

Male B6 mice weighing 18–22 g were pretreated with C12 solution (15 mg/kg) by intravenous (i.v.) injection for 15 min and then 200 µL of LPS (10 mg/kg), dissolved in saline (0.9% NaCl), was i.v. injected. Negative control animals received only a similar volume (200 µL) of saline. Mice were anesthetized with diethyl ether and sacrificed at the indicated time after LPS injection. Blood samples were taken from the right ventricle orbital veniplex using a heparinized syringe with a needle. Liver and plasma were flash frozen in liquid nitrogen and stored at −80°C until analysis.

### Determination of TNF-α and IL-6

After treatment of mice with C12 and LPS, the TNF-α and IL-6 levels in plasma were determined with an enzyme-linked immunosorbent assay (ELISA) kit (Bioscience, San Diego, CA) according to the manufacturer's instructions. The total amount of the inflammatory factor in the media was normalized to the total protein quantity of the viable cell pellets.

### Real-time quantitative PCR

Cells or liver tissues (50–100 mg) were homogenized in TRIZOL (Invitrogen, Carlsbad, CA) or prepared with a PARIS kit (Ambion, Austin, TX) for extraction of RNA according to each manufacturer's protocol. Both reverse transcription and quantitative PCR were carried out using a two-step M-MLV Platinum SYBR Green qPCR SuperMix-UDG kit (Invitrogen, Carlsbad, CA). Eppendorf Mastercycler ep realplex detection system (Eppendorf, Hamburg, Germany) were used for q-PCR analysis. The primers of genes including iNOS, COX-2, TNF-α, IL-6, prostaglandin synthetase (PGES), IL-1β and β-actin were synthesized by Invitrogen. The primer sequences used are shown as follows: mouse TNF-α sense primer: 5′-TGGAACTGGCAGAAGAGG-3′, Mouse TNF-α antisense primer: 5′-AGACAGAAGAGCGTGGTG-3′; mouse IL-6 sense primer: 5′-GAGGATACCACTCCCAACAGACC-3′, mouse IL-6 antisense primer: 5′-AAGTGCATCATCGTTGTTCATACA-3′; mouse COX-2 sense primer: 5′-TGGTGCCTGGTCTGATGATG-3′, mouse COX-2 antisense primer: 5′-GTGGTAACCGCTCAGGTGTTG-3′; mouse PGES (mPEGS) sense primer: 5′-ACACTGCTGGTCATCAAG-3′, mouse PGES antisense primer: 5′-GGTCACTCCTGTAATACTGG-3′; mouse iNOS sense primer: 5′-CAGCTGGGCTGTACAAACCTT-3′; mouse iNOS antisense primer: 5′-CATTGGAAGTGAAGCGTTTCG-3′; mouse IL-1β sense primer: 5′-ACTCCTTAGTCCTCGGCCA-3′, mouse IL-1β antisense primer: 5′-CCATCAGAGGCAAGGAGGAA-3′; mouse β-actin sense primer: 5′-TGGAATCCTGTGGCATCCATGAAAC-3′, mouse β-actin antisense primer: 5′-TAAAACGCAGCTCAGTAACAGTCCG-3′. The amount of each gene was determined and normalized by the amount of β-actin.

### Lung histopathology

Two days after LPS injection (10 mg/kg, i.v.), the lungs of B6 mice C12-pretreated (15 mg/kg, i.v.) or control were distended by infusion of 10% formalin into the trachea at a pressure of 15 cm H_2_O for 2 h, and fixed in 10% formalin for 24 h. Formalin-fixed lungs were embedded in paraffin, sectioned and stained with hematoxylin and eosin (H&E). To estimate the extent of damage, the specimen was observed under a light microscope.

### LPS-induced inflammatory mortality in B6 mice

Male B6 mice weighing 18–22 g were pretreated with C12 (15 mg/kg) by i.v. injection 15 min before the i.v. injection of LPS (20 mg/kg). Control animals received a similar volume (10 mL/kg) of saline. Body weight change and mortality were recorded for 7 days.

### Vascular permeability assay in ICR mice

Male ICR mice weighing 18–22 g were pretreated with dexamethasone (5 mg/kg) or C12 (5 or 15 mg/kg) intraperitoneally. Control animals received a similar volume (200 µL/kg) of saline. After 15 min, 200 µL of 10% azovan blue solution in saline was i.v.-injected and 300 µL of acetic acid (0.7%) was i.p.-injected, respectively. Then, 30 min after acetic acid injection, the peritoneal fluid was collected in sterile and heparinized PBS (1 mL) solution. The total of the collection was evaluated by optical density assay (620 nm).

### Carrageenan-induced paw oedema in mice

Male ICR mice weighing 18–22 g were divided into four groups (n = 6 in each), that were submitted with respect to the treatment by the i.p. route with saline, Dexamethasone (5 mg/kg, Xianju Pharma, Zhejiang, China) or C12 (5 or 15 mg/kg), 15 min before oedema induction by subcutaneous injection of carrageenan (300 µg/paw) in the right paw. The left paw was injected with an equal volume of saline as a negative control, and both paws were monitored by a procedure similar to that described elsewhere [19]. The paw oedema was measured by a electronic micrometer and calculated by the difference between paw volumes 4 h after paw oedema induction.

### Chemically-induced inflammatory abdominal constrictions in mice

Abdominal constrictions to the i.p. injection of acetic acid (0.6%) were monitored. The procedure was similar to that previously described by de Campos et al. (18). Male ICR mice weighing 18–22 g were pretreated with Dexamethasone (5 mg/kg) or C12 (5 or 15 mg/kg) intraperitoneally 15 min before the injection of acetic acid. Control animals received a similar volume (10 mL/kg) of saline. After challenge, pairs of mice were placed in separate boxes, and the number of abdominal constrictions was cumulatively counted.

### Formalin-induced inflammatory nociception in mice

The procedure used was similar to that previously described by Correa and Calixto [30]. Male ICR mice weighing 18–22 g were pretreated intraperitoneally with C12 (5 or 15 mg/kg) or Dexamethasone (5 mg/kg) 15 min before pain induction. Control animals received only saline. 20 µL of 2.5% formalin in PBS was injected under the paw surface of the right hind paw. The mice (control and treated groups) were observed simultaneously from 0–30 min following formalin injection. The amount of time spent licking the injected paw was timed with a chronometer and was considered indicative of pain.

### Statistical analysis

The results are presented as means±SD. The statistical significance of differences between groups was obtained by means of analysis of variance followed by the post-test of comparison. P values less than 0.05 (*p*<0.05) were considered indicative of significance. Nitrite determination and cell viability were performed in triplicate. All experiments were repeated at least three times.

## Supporting Information

Figure S1 **Toxicity assay of C12.** Male ICR mice weighing 18–22 g were randomly seperated into 4 groups (n = 10 in each group) and were treated with C12 at 5, 10, 20, or 40 mg/kg by i.p. administration. Survival of the mice was monitored for 14 days.
(DOC)

Text S1 **The toxicity of C12 in vivo.**
(DOC)
