# Supplementary material for: A Novel Compound C12 Inhibits Inflammatory Cytokine Production and Protects from Inflammatory Injury In Vivo
Source: PLoS One. 2011 Sep 8;6(9):e24377. doi: 10.1371/journal.pone.0024377 (PMC3169595; doi:10.1371/journal.pone.0024377)
Supplement: Figure S1 — Toxicity assay of C12. Male ICR mice weighing 18–22 g were randomly seperated into 4 groups (n = 10 in each group) and were treated with C12 at 5, 10, 20, or 40 mg/kg by i.p. administration. Survival of the mice was monitored for 14 days. (DOC) [file pone.0024377.s001.doc]

**Figure S1.** Toxicity assay of C12. ICR mice weighing 18-22g were randomly seperated into 4 groups (n=10 in each group) and were treated with C12 at 5, 10, 20, or 40 mg/kg by i.p. administration. Survival of the mice was monitored for 14 days.
