# Supplementary material for: A Novel Compound C12 Inhibits Inflammatory Cytokine Production and Protects from Inflammatory Injury In Vivo
Source: PLoS One. 2011 Sep 8;6(9):e24377. doi: 10.1371/journal.pone.0024377 (PMC3169595; doi:10.1371/journal.pone.0024377)
Supplement: Text S1 — The toxicity of C12 in vivo. (DOC) [file pone.0024377.s002.doc]

**Supporting information**

**A novel compound C12 inhibits inflammatory cytokine production and protects from inflammatory injury *in vivo***

Yi Wang, Congcong Yu, Yong Pan, Jianling Li, Yali Zhang, Faqing Ye, Shulin Yang, Hui Zhang, Xiaokun Li, Guang Liang

**Text S1: *The toxicity of C12 in vivo***

**Materials and Methods:**

Male ICR mice weighing 18-22 g were randomly separated into 4 groups (with n=10), that were submitted with respect to the treatment by intraperitoneal (i.p.) route with C12 (5, 10, 20, or 40 mg/kg). Mortality was monitored for 14 days.

**Results:**

As shown in Figure S1, by day 14, all mice in treatment groups of all doses survived. Given this result and the studies *in vitro*, mice were administered with C12 at 15 mg/kg (i.p.) or 10 and 30 mg/kg (i.v.) in the following *in vivo* experiments.
